# Supplementary material for: Gamma oscillations point to the role of primary visual cortex in atypical motion processing in autism
Source: PLoS One. 2023 Feb 13;18(2):e0281531. doi: 10.1371/journal.pone.0281531 (PMC9925089; doi:10.1371/journal.pone.0281531)
Supplement: S1 Fig — (HTML) [file pone.0281531.s001.html]

Supplementary Figure 1


Supplementary Figure 1

Filter by tags

- Select all
- ---
- Subject-1
- Subject-10
- Subject-11
- Subject-12
- Subject-13
- Subject-14
- Subject-15
- Subject-16
- Subject-17
- Subject-18
- Subject-19
- Subject-2
- Subject-20
- Subject-21
- Subject-22
- Subject-23
- Subject-24
- Subject-25
- Subject-26
- Subject-27
- Subject-28
- Subject-29
- Subject-3
- Subject-30
- Subject-31
- Subject-32
- Subject-33
- Subject-34
- Subject-35
- Subject-36
- Subject-37
- Subject-38
- Subject-39
- Subject-4
- Subject-40
- Subject-41
- Subject-42
- Subject-43
- Subject-44
- Subject-45
- Subject-46
- Subject-47
- Subject-48
- Subject-49
- Subject-5
- Subject-50
- Subject-51
- Subject-52
- Subject-53
- Subject-54
- Subject-55
- Subject-56
- Subject-57
- Subject-58
- Subject-59
- Subject-6
- Subject-60
- Subject-61
- Subject-62
- Subject-63
- Subject-64
- Subject-65
- Subject-66
- Subject-67
- Subject-68
- Subject-7
- Subject-8
- Subject-9

##### Table of contents

Description
Control
Control
Control
Control
Control
Control
Control
Control
Control
Control
Control
Control
Control
Control
Control
Control
Control
Control
Control
Control
Control
ASD
ASD
ASD
ASD
ASD
ASD
ASD
ASD
ASD
ASD
ASD
ASD
ASD
ASD
ASD
ASD
ASD
ASD
Control
Control
Control
Control
Control
Control
Control
Control
ASD
ASD
ASD
ASD
ASD
ASD
ASD
ASD
ASD
ASD
Control
Control
Control
Control
Control
ASD
ASD
ASD
ASD
ASD
ASD

Description

Control

Subject-1

Control

Subject-2

Control

Subject-3

Control

Subject-4

Control

Subject-5

Control

Subject-6

Control

Subject-7

Control

Subject-8

Control

Subject-9

Control

Subject-10

Control

Subject-11

Control

Subject-12

Control

Subject-13

Control

Subject-14

Control

Subject-15

Control

Subject-16

Control

Subject-17

Control

Subject-18

Control

Subject-19

Control

Subject-20

Control

Subject-21

ASD

Subject-22

ASD

Subject-23

ASD

Subject-24

ASD

Subject-25

ASD

Subject-26

ASD

Subject-27

ASD

Subject-28

ASD

Subject-29

ASD

Subject-30

ASD

Subject-31

ASD

Subject-32

ASD

Subject-33

ASD

Subject-34

ASD

Subject-35

ASD

Subject-36

ASD

Subject-37

ASD

Subject-38

ASD

Subject-39

Control

Subject-40

Control

Subject-41

Control

Subject-42

Control

Subject-43

Control

Subject-44

Control

Subject-45

Control

Subject-46

Control

Subject-47

ASD

Subject-48

ASD

Subject-49

ASD

Subject-50

ASD

Subject-51

ASD

Subject-52

ASD

Subject-53

ASD

Subject-54

ASD

Subject-55

ASD

Subject-56

ASD

Subject-57

Control

Subject-58

Control

Subject-59

Control

Subject-60

Control

Subject-61

Control

Subject-62

ASD

Subject-63

ASD

Subject-64

ASD

Subject-65

ASD

Subject-66

ASD

Subject-67

ASD

Subject-68

Created on мая 03, 2022 via MNE-Python 0.24.0
