# Supplementary material for: Gamma oscillations point to the role of primary visual cortex in atypical motion processing in autism
Source: PLoS One. 2023 Feb 13;18(2):e0281531. doi: 10.1371/journal.pone.0281531 (PMC9925089; doi:10.1371/journal.pone.0281531)
Supplement: S1 Table — (DOCX) [file pone.0281531.s003.docx]

Supplementary table. Spearman correlations of psychometric variables (MPI IQ, SRS) with MEG and psychophysical parameters in children with ASD.

|  | GR frequency* | | | Log10 GR power | | | GRSI* | Log10 Thresholds** | |
| --- | --- | --- | --- | --- | --- | --- | --- | --- | --- |
|  | Slow | Medium | Fast | Slow | Medium | Fast |  | Large | Small |
| MPI IQ | N = 34  R = -0.35 p = 0.04 | N = 30  R = -0.42 p = 0.02 | N = 22  R = -0.17  p = 0.44 | N = 42  R = 0.12  p = 0.44 | N = 42  R = 0.15  p = 0.34 | N = 42  R = -0.00 p = 0.99 | N = 34  R=- 0.02  p = 0.90 | N = 33  R = 0.00 p = 0.99 | N = 33  R= -0.07 p = 0.69 |
| SRS *** | N = 30  R= -0.11 p = 0.53 | N = 27  R = 0.01 p = 0.53 | N = 20  R = -0.11 p = 0.53 | N = 38  R = 0.26  p = 0.11 | N = 38  R = 0.17 p = 0.29 | N = 38  R = 0.13 p = 0.48 | N = 30  R= -0.24 p = 0.21 | N = 29  R= -0.22  p = 0.24 | N = 29  R= -0.01  p = 0.94 |

Note: Gamma response (GR) frequency and power were corrected for age. Slow/medium/fast correspond to motion velocity condition in the MEG experiment. P values are uncorrected for multiple comparisons.

* GR frequency and GRSI were estimated only for reliable GRs.

** Psychometric thresholds were measured in 33 of 42 boys with ASD

*** SRS data were available for 38 of 42 boys with ASD
