# Supplementary material for: Gamma oscillations point to the role of primary visual cortex in atypical motion processing in autism
Source: PLoS One. 2023 Feb 13;18(2):e0281531. doi: 10.1371/journal.pone.0281531 (PMC9925089; doi:10.1371/journal.pone.0281531)
Supplement: Attachment — Submitted filename: PONE-D-22-28959_review_report.docx [file pone.0281531.s005.docx]

The focus of the present study, titled “Gamma oscillations point to the role of primary visual cortex in atypical motion

processing in autism” from Orekhova et al., was the relationship between stimulation-related modulations of MEG gamma oscillations and perception of visual motion in children with ASD.

Considering how for some children with ASD, it is difficult to tolerate MEG and MRI data collection procedures, this work can give useful data.

After reading this work I have no specific requests for the authors and I consider the work done so far good for publication.
