# Supplementary material for: Gamma oscillations point to the role of primary visual cortex in atypical motion processing in autism
Source: PLoS One. 2023 Feb 13;18(2):e0281531. doi: 10.1371/journal.pone.0281531 (PMC9925089; doi:10.1371/journal.pone.0281531)
Supplement: Attachment — Submitted filename: Response to Reviewers.docx [file pone.0281531.s006.docx]

**Responses to Editor’s comments**

The reviewer were mostly positive. There are some minor changes, esp. in the figures, for instance Fig 1 and 2 may include some raw data or event related activity in time domain which will help to make sense of the observed spectral changes. Fig 1a, middle panel is not clear.

Also important info on the figure conventions and panels are missing in the captions. For instance, Figure 6 includes many panels (columns) and can be improved by including titles to the panels, and referring back to them in the text. 

We are grateful to the Editor and Reviewers for the positive evaluation of our work.

As suggested, we revised figure 1 by including the raw data. We also made captions over all the panels, which, we hope, made the content easier to understand. Since Figure 2 presents information similar to that in Figure 1b (right-hand panels), we believe that the inclusion of raw data in this figure may be redundant. We marked all panels in figure 6 by letters of the alphabet and refer to them in the text.

**Responses to Reviewer’s comments**

Reviewer #1: The focus of the present study, titled “Gamma oscillations point to the role of primary visual cortex in atypical motion processing in autism” from Orekhova et al., was the relationship between stimulation-related modulations of MEG gamma oscillations and perception of visual motion in children with ASD.

Considering how for some children with ASD, it is difficult to tolerate MEG and MRI data collection procedures, this work can give useful data.

After reading this work I have no specific requests for the authors and I consider the work done so far good for publication.

We are grateful to the reviewer for the positive evaluation of our work.

Reviewer #2:

We are grateful to the reviewer for taking the time to review our manuscript and provide comments.

1. In the material and methods authors indicated that only TD and ASD boys were employed, I just suggest to explain in a brief sentence, why girls are not included (male to female ratio in ASD).

We added the following text to the Methods section (lines 141-145):

*We limited our sample to males because the ratio of males to females among people with ASD is very high (~4/1) [51], and the relatively small sample size of our study would not have allowed us to analyze female participants with ASD as a separate group. On the other hand, the putative differences in neural excitability between males and females with ASD [52] preclude combining them into a single sample.*

2. Even if well explained in the text, I suggest to add a more detailed description also in figure legend 1.

Thank you for this suggestion. We added more information to the legend.

*Fig 1. Overview of the data collection and analysis pipelines. (a) Psychophysical experiment. Large or small gratings moving left or right were presented for a short period of time. The subject had to indicate the direction of motion by pressing the corresponding button. In case of a wrong response the exposure time increased, while in case of two correct responses in a row it decreased (‘one-up—two-down staircase’). The minimum time required for the subject to detect direction of motion (i.e. duration threshold) of small and large gratings was defined as the average exposure time at all but the first two ‘reversals’ of the corresponding staircase. (b) MEG experiment. The subject was presented with circular gratings drifting toward the center at different speeds. Brain activity corresponding to baseline and visual stimulation intervals was localized using LCMV beamforming. Stimulation-related changes in gamma power (gamma response) were calculated as (stimulation-baseline)/baseline. The peak frequency and power of gamma response (GR) were analyzed at ‘maximal sources’ in the visual cortex. For a detailed description of the experimental procedures and analysis, see the Methods section.*
